# Hydrogel composite containing azelaic acid and tea tree essential oil as a therapeutic strategy for *Propionibacterium* and testosterone-induced acne

**DOI:** 10.1007/s13346-021-01092-4

**Published:** 2021-11-15

**Authors:** Alpna Bisht, Chetna Hemrajani, Charul Rathore, Tania Dhiman, Rajan Rolta, Navneet Upadhyay, Prakriti Nidhi, Gaurav Gupta, Kamal Dua, Dinesh Kumar Chellappan, Kamal Dev, Anuradha Sourirajan, Apala Chakraborty, Alaa A. A. Aljabali, Hamid A. Bakshi, Poonam Negi, Murtaza M. Tambuwala

**Affiliations:** 1 School of Pharmaceutical Sciences, Shoolini University of Biotechnology and Management Sciences, Solan, India 173 212; 2 School of Applied Sciences and Biotechnology, Shoolini University of Biotechnology and Management Sciences, Solan, Himachal Pradesh India; 3 School of Pharmacy, Suresh Gyan Vihar University, Jagatpura, Mahal Road, Jaipur, India; 4 Centre for Inflammation, Centenary Institute, Royal Prince Alfred Hospital, Missenden Rd, Sydney, NSW 2050 Australia; 5 Discipline of Pharmacy, Graduate School of Health, University of Technology Sydney, Ultimo, NSW 2007 Australia; 6 Priority Research Centre for Healthy Lungs, School of Biomedical Sciences and Pharmacy, Hunter Medical Research Institute (HMRI, University of Newcastle, Callaghan, NSW 2308 Australia; 7 Department of Life Sciences, School of Pharmacy, International Medical University, 57000 Kuala Lumpur, Malaysia; 8 Department of Pharmaceutical Technology, Jadavpur University, 188, Raja S.C Mallick Road, Jadavpur, Kolkata, India 700032; 9 Department of Pharmaceutics and Pharmaceutical Technology, Faculty of Pharmacy, Yarmouk University, Irbid, 21163 Jordan; 10 School of Pharmacy and Pharmaceutical Science, Ulster University, Coleraine, BT52 1SA Northern Ireland UK

**Keywords:** *P. acne*, Agar well diffusion method, Broth microdilution assay, Rheology, Skin permeation, Testosterone acne model

## Abstract

Azelaic acid (AzA) is a USFDA bioactive prescribed against *acne vulgaris*. It possesses delivery challenges like poor aqueous solubility, low skin-penetrability, and dose-dependent side effects, which could be overcome by its synergistic combination with tea tree oil (TTO) as a microemulsion (ME)-based hydrogel composite. AzA-TTO ME was prepared to employ pseudo-ternary phase diagram construction. The best AzA-TTO ME was of uniform size (polydispersity index < 0.7), nano-range (~357.4 ± 2% nm), transmittance (> 90%), and negative zeta potential (−1.42 ± 0.25% mV) values. ME hydrogel composite with optimum rheological and textural attributes showed better permeation, retention, and skin-compliant characteristics, vis-a-vis marketed formulation (Aziderm™) when evaluated in Wistar rat skin. In vitro antibacterial efficacy in bacterial strains, i.e., *Staphylococcus aureus*, *Propionibacterium acne*, and *Staphylococcus epidermidis*, was evaluated employing agar well plate diffusion and broth dilution assay. ME hydrogel has shown an increase in zone of inhibition by two folds and a decrease in minimum inhibitory concentration (MIC) by eightfold against *P. acnes* vis-a-vis AzA. Finally, ME hydrogel composite exhibited a better reduction in the papule density (93.75 ± 1.64%) in comparison to Aziderm™ 72.69 ± 4.67%) on acne as developed in rats by inducing testosterone. Thus, the developed AzA-TTO ME hydrogel composite promises an efficacious and comparatively safer drug delivery system for the topical therapy of *acne vulgaris*.

**Graphical abstract:**

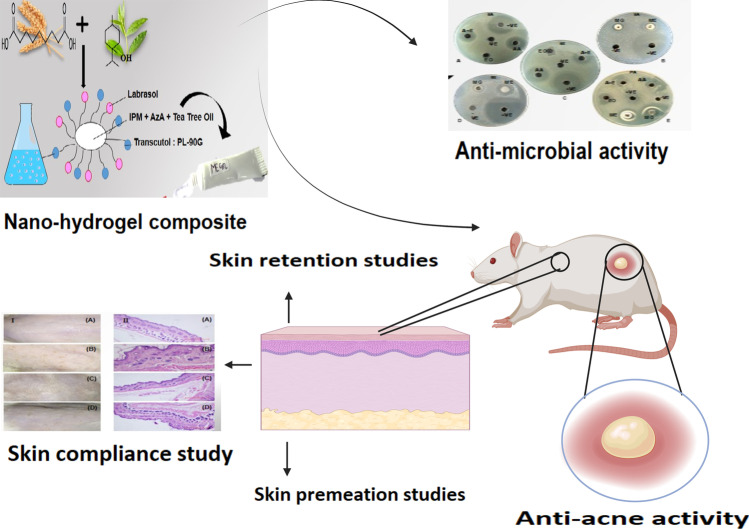

## Introduction

Acne vulgaris is a severe, multifactorial disorder of skin generally rigorous over pilosebaceous units. Listed amongst various common, occurring dermatological conditions worldwide, it inflicts profound effects on physiological health, causing depression, social ideation, anxiety, psychosomatic indication, sham, embarrassment, and social inhibition. The pathogenesis of acne vulgaris includes disturbed sebaceous gland function, with hypersecretion of sebum and alteration in its fatty acid compositions, follicular hyper-keratinization, hormonal dysregulation, neuropeptides induced inflammation, and also the dysfunction of both innate and adaptive immune systems [1]. Trapped sebum in the pilosebaceous glands of regular skin produces a substrate that helps grow bacterial flora such as Staphylococcus aureus, Staphylococcus epidermidis *Propionibacterium acne* and leads to the constitution of comedones and other inflammatory lesions are formed [2, 3].

The primary choice for the treatment of mild to moderate acne is topical therapy. The topical therapy of *acne vulgaris* mainly depends on retinoids, including tretinoin, adapalene, tazarotene, isotretinoin, and fenretinide. At the same time, topical antibiotics including clindamycin, erythromycin, clarithromycin, azithromycin, and nadifloxacin are also being used. These drugs are associated with many side effects and have developed bacterial resistance over the last decades [4]. Researchers are currently focusing on newer promising herbal agents, including isolated molecules like azelaic acid (AzA) and essential oils obtained from the plant *Melaleuca alternifolia* [5, 6], having more minor side effects and bacterial resistance.

AzA is a naturally obtained saturated dicarboxylic acid mostly available in wheat, barley, and rye. It possesses antibacterial, keratolytic, comedolytic, and anti-inflammatory actions. This drug is used topically to reduce inflammation connected with acne vulgaris and rosacea. AzA has not developed any bacterial resistance and comprises significantly fewer side effects than the other therapeutic agents [6]*.* Despite having several benefits of AzA, a higher dose (10% w/w or 20% w/w of AzA) is required in formulation to reach the desired therapeutic effects due to its poor solubility and low skin penetrability. Thus, dose-dependent adverse effects such as skin irritation, dryness, scaling, peeling, and erythema warrant a novel strategy for effective delivery. Essential oil like tea tree oil (TTO) is used for many years to treat acne vulgaris against *Propionibacterium acne*, *Staphylococcus aureus*, and *Staphylococcus epidermidis* like bacteria. It is obtained from steam distillation of follicular leaves, an Australian indigenous plant of *Melaleuca alternifolia*. Skin commensals: *S. aureus*, *S. epidermidis*, and *P. acnes* possess pathogenic potential in acne skin, triggering proinflammatory cytokine/chemokine production in infected keratinocytes. Thus, to reduce the dose-dependent side-effects of AzA and achieve synergistic effects, it can be co-formulated with TTO, which is very effective against acne vulgaris.

Nanocarriers are gaining wide acceptance as drug delivery systems, and in the past, many attempts have been made to deliver AzA topically by applying nanotherapeutics. This includes the application of liquid crystal systems [7, 8], liposomes [9], ethosomes [10], proteasomes [11], nanoparticles [12], leciplex, invasions [13], nanostructured lipid carriers [14], nanoemulsion [15], and microemulsion [16], which are found to be efficient to deliver AzA across the skin. TTO has also been formulated as nanoemulsion [17], liposome [18], ethosome [19], microsponges [20], nanoparticles [21], and microcapsules [22].

AzA and TTO can be co-delivered in the form of a thermodynamically stable microemulsion (ME) system. ME is an anisotropically transparent system consisting of two immiscible liquids, i.e., oil and water, stabilized by surfactant molecules. ME presents several benefits like ease of preparation, increased drug solubilization, permeation enhancement, and increased thermodynamic activity of a drug on the skin. Further, this drug delivery system shows a better drug release profile and improved skin permeation and targeting. The microdroplets of ME could reach close contact with the skin and provide a larger surface area, followed by an improved absorption rate for the drug. Further, MEs’ continuous and spontaneous interfacial fluctuation allows a higher rate of drug mobility and diffusion [16]. In this study, ME formulations of AzA and TTO were optimized employing pseudo-ternary phase diagrams and investigated for their effectiveness against *S. epidermidis*, *S. aureus*, and *P. acnes* evaluated employing agar well plate diffusion and broth dilution assay.

Carbopol 934, a polymer of acrylic acid cross-linked in the company of polyalkenyl ethers or divinyl glycol, was employed to transform AzA and TTO integrated ME hydrogel composite [23]. The three-dimensional polymeric network presents excellent biocompatibility, hydration, ease of fabrication, high swelling ability, stabilizing ability, desirable rheological and textural attributes, and can be potentially exploited in the topical application without disturbing the ME architecture [24]. The skin permeability, and absorption potential, of the ME and ME hydrogel composite, was also compared with the marketed formulation (Aziderm™) employing the Wistar rat skin. Finally, skin safety was assessed in Wistar rats, while anti-acne effectiveness was evaluated in Swiss albino mice employing a testosterone-induced acne model.

## Materials and methods

### Materials

AzA was purchased from TCI Chemicals Pvt. Ltd., Chennai, India. TTO (*Melaleuca alternifolia*) was purchased from Allin Exporters, Noida, India. Phospholipipon 90G (PL-90G) was supplied ex gratis by Phospholipid GmbH, Germany. Dimethyl sulfoxide (DMSO), isopropyl myristate (IPM), Tween 80, Carbopol 934, PEG 400, triethanolamine (TEA), acetonitrile (ACN), sodium dihydrogen orthophosphate, and orthophosphoric acid were purchased from Loba Chemie Pvt. Ltd., Mumbai, India. Absolute ethanol was purchased from Changshu Hongsheng Fine Chemical Co. Ltd., Changshu, China. Nutrient broth, Agar, Brain heart infusion (BHI), Erythromycin, and Resazurin Dye were purchased from Himedia Labs Pvt. Ltd., Kolkata, India. In the study, the commercial formulation of azelaic acid was Aziderm 10% w/w gel (Micro labs Ltd. Bangalore, India) procured from a local drug house. Ultrapure water prepared by Micropore™ assembly installed at Shoolini University, Solan, was used throughout the study.

The present investigation was conducted as per the guidelines of CPCSEA (Committee for controlling and supervising experiments on animals). The Institutional Animal Ethical Committee duly approved the experimental protocol of Shoolini University (IAEC/SU/09/18). Male Swiss albino mice and Wistar rats used in the current study were obtained from the central animal house of Shoolini University Solan (HP).

### Methods

#### RP-HPLC Instrumentation and chromatographic conditions for determining AzA

The analysis of AzA was conducted using an HPLC system (Agilent Technology 1200 series, Chandigarh, India) connected with a double beam UV spectrophotometer (Systroni2202, India Pvt. Ltd., Mumbai, India), pressure controlled by prominence pump and operated by EZChrom software. An innoval C18 HPLC column (250 × 4.6 mm, particle size 5 µm) was used for the separation, and the column was maintained at 25 °C. Two different mobile phase solvents for pumps were (50:50) sodium di-hydrogen orthophosphate (NaH_2_PO_4_; pH 3.5; 50 mM) pump A, and ACN for pump B. Filtration of the mobile phase were done through a 0.45 µm nylon membrane filter followed by ultrasonication for 30 min. Before the injection, samples were filtered through the nylon syringe filter (0.22 µm). A mixture of Sodium dihydrogen orthophosphate and ACN (1:1) v/v was used as a mobile phase in gradient mode. The flow rate was maintained at 0.6 mL/min. Ten-microliter volume of sample was injected with a runtime of 30 min during analysis, maintaining the PDA detector at 321 nm. The detection wavelength for the analysis was 250 nm [25].

#### Construction of PTPD

IPM was selected as the oil phase for constructing all pseudo-ternary phase diagram (PTPD) based on the solubility profile of AzA in the different components and the miscibility of TTO with oil. Tween 80 and Labrasol were selected as surfactants in this study. At the same time, Transcutol P and PL-90G- ethanol (Ratio 1:10) were selected as co-surfactants (CS). Diverse combinations of surfactants-CS mixture (Smix ratio), IPM, and water were taken, and an aqueous titration method was employed to investigate the phase behavior (Table 1). The first four ternary phase diagrams were constructed usingLabrasol as a surfactant and Transcutol P and PL-90G: ethanol as CS. Transcutol P and PL-90G ratio was fixed as 1:1, while Smix ratio was altered at 1:1, 2:1, 3:1, and 4:1 for the PTPD 1–4 MEs, respectively. In the PTPD 5, only Labrasol was employed, without any CS. However, in the PTPD 6, only Transcutol P was employed as CS, and the ratio of Labrasol: Transcutol P (Smix), was kept as 1:1. In PTPD 7 and 8, CS employed were Transcutol P and PL-90G: ethanol in the ratio 1:1, while Smix ratio (Tween 80: CS) was altered at 1:2 and 2:1, respectively. In PTPD 9, only PL-90G: ethanol was used as CS, Tween 80 as a surfactant, and the Smix ratio was 1:1. In PTPD 10 and 11, only Transcutol P was employed CS, Tween 80 as a surfactant, and the Smix ratio was altered at 1:1 and 2:1, respectively. In PTPD 12, Transcutol P and PL-90G-ethanol were used at Smix ratio 1:1, and no surfactant was used (Table 1). The area under the triangular phase diagrams denoting ME was plotted using computer software (PCP Disso v2.08, Pune, India) [26].

**Table 1:** Components and their ratios employed to construct a different pseudo-ternary phase diagram

**S.NO**	**Oil**	**Smix**		
		**Surfactant**	**Cosurfactant**	**Ratio**
1	IPM	Labrasol	Transcutol P:(PL-90G + ethanol (1:10)) (1:1)	1:1
2	IPM	Labrasol	Transcutol P:(PL-90G + ethanol (1:10)) (1:1)	2:1
3	IPM	Labrasol	Transcutol P:(PL-90G + ethanol (1:10)) (1:1)	3:1
4	IPM	Labrasol	Transcutol P:(PL-90G + ethanol (1:10)) (1:1)	4:1
5	IPM	Labrasol	-	_
6	IPM	Labrasol	Transcutol P	1:1
7	IPM	Tween 80	Transcutol P:(PL-90G + ethanol (1:10)) (1:1)	1:1
8	IPM	Tween 80	Transcutol P:(PL-90G + ethanol (1:10)) (1:1)	2:1
9	IPM	Tween 80	PG + ethanol (1:10)	1:1
10	IPM	Tween 80	Transcutol P	1:1
11	IPM	Tween 80	Transcutol P	2:1
12	IPM	_	Transcutol P:(PL-90G + ethanol (1:10)) (1:1)	_

*IPM* isopropyl myristate, *PL-90G* Phospholipon 90G, *Smix* surfactant:Co surfactant ratio

#### Preparation of microemulsions (ME1-ME24)

Two different combinations were optimized from the ME region of each PTPD individually. AzA and TTO were dissolved in the mixture of oil and Smix (IPM/Labrasol/Tween 80/Transcutol P/PL-90G) with sonication, then the required amount of water was added based on values obtained from corresponding PTPD. This was followed by mixing via vortex shaker to obtain the formulation, an anisotropic and transparent solution. Table 2 lists all the prepared MEs and their compositions (ME1-ME24) [26].

**Table 2:** Selection of ME compositions based on the drug solubility

**S. no**	**Oil (%)**	**Smix (%)**	**Water (%)**	**Drug (mg/mL)**
**1**	IPM	Labrasol:(Transcutol P:( PL-90G:ethanol (1:10)) (1:1)) 1:1		
a	55	30	10	40
b	60	20	15	40
**2**	IPM	Labrasol:Transcutol P:(PL-90G:ethanol (1:10)) (1:1)2:1		
a	35	55	5	90
b	45	40	5	80
**3**	IPM	Labrasol:Transcutol P:( PL-90G:ethanol (1:10)) (1:1)3:1		
a	40	45	10	100
b	35	55	5	100
**4**	IPM	Labrasol:Transcutol P:( PL-90G:ethanol (1:10)) (1:1)4:1		
a	30	60	5	70
b	40	50	5	60
**5**	IPM	Labrasol		
a	25	65	5	40
b	35	50	10	30
**6**	IPM	Labrasol:Transcutol P 1:1		
a	60	25	10	40
b	55	20	20	40
**7**	IPM	Tween 80:Transcutol P:(PL-90G + ethanol (1:10)) (1:1)1:1		
a	45	40	10	80
b	30	60	5	80
**8**	IPM	Tween 80:Transcutol P:(PL-90G + ethanol (1:10)) (1:1)2:1		
a	45	40	10	40
b	40	50	5	60
**9**	IPM	Tween 80: PL-90G + ethanol (1:10) 1:1		
a	20	70	5	30
b	25	60	10	40
**10**	IPM	Tween 80:Transcutol P 1:1		
a	30	60	5	50
b	35	55	5	50
**11**	IPM	Tween 80:Transcutol P 2:1		
a	25	60	10	50
b	35	50	10	50
**12**	IPM	Transcutol P:( PL-90G:ethanol (1:10)) (1:1)		
a	70	15	10	30
b	70	10	15	10

TTO was used 5% in all formulations

*IPM* isopropyl myristate, *PL-90G* phospholipid 90G, *Smix* surfactant:cosurfactant

#### Selection of MEs for characterization (MET 1–6)

The ME composition has more solubility for both the active ingredients individually, i.e., AzA and TTO were selected for further characterization.

### Characterization of AzA-TTO-loaded ME

#### Micromeretics

The polydispersity index (PDI) and the prepared MEs’ droplet size were determined by dynamic light scattering (DLS) using a Malvern Zeta-sizer analyzer installed at the Department of Chemistry Panjab University, Chandigarh. DLS, also known as photon correlation spectroscopy, measures the time correlation as a function of the scattered intensity. Calculation of the diffusion coefficient of the ME droplets was done by correlating reduction in correlation function with displacement time. The hydrodynamic radius of ME droplets was calculated from the diffusion coefficient. The mean value of the three repeated measurements was considered as the final measurement [27].

### Selection of optimized ME

The optimized ME was selected from the selected MEs (ME1-6) based on the least particle size and PDI values.

#### TEM analysis

The prepared ME was characterized for morphology employing transmission electron microscope (TEM) installed at Central Instrumentation Laboratory (CIL), Panjab University, Chandigarh. The negative staining of the selected ME system (MET-6) was performed using phosphotungstic acid (1% w/v)aqueous solution, subsequently dried on a microscopic carbon-coated grid. The prepared grid was observed under a microscope at appropriate magnification [26].

#### Zeta potential

Zetasizer analyzed the zeta potential of selected MET-6- LS13320 (DelsaTMNano C Beckman colter) installed at the Institute of Pharmaceutical Sciences, Panjab University, Chandigarh. The final result was reported as mean ± SD (*n* = 3) [28].

#### % Transmittances

Transparency of the selected ME formulation (MET-6) was determined by measuring the % transmittance at 650 nm by using a UV spectrophotometer taking purified water as blank [29].

### Preparation of AzA-TTO co-loaded hydrogel composite

Incorporating ME in the polymeric hydrophilic carbopol fibers can form the composite hydrogel matrix, providing an architecture that mimics the microenvironment of the skin. Such conversion also facilitates the ease of application, adhesion, and penetration of AzA and TTO from the ME globules. To prepare AzA-TTO ME hydrogel composite, 10% (w/w) aqueous carbopol resin solution was prepared. Triethanolamine was used as a neutralizing agent. The gel composite was adjusted to the desired pH of 7 by using the optimum amount of triethanolamine. The hydrogel composite was allowed to swell at room temperature for 24 h. The AzA-TTO ME was then incorporated in 10% by weight of neutralized carbopol® 934 hydrogel, resulting in the final percentage of carbopol in the ME as 2% w/w.

#### Organoleptic properties

Organoleptic properties like transparency, grittiness, feel, and tackiness was observed visually in the prepared ME hydrogel. The transparency of the hydrogel composite was observed against a white light source. The other characteristics of the ME hydrogel were assessed by rubbing it gently between the thumb and forefinger.

#### pH Measurement

The pH of the drug-loaded ME and ME hydrogel sample (undiluted) was measured by dipping the electrode of a calibrated pH meter into the sample at 25 °C.

All measurements were done in triplicate, and the value was expressed in mean ± SD [30].

#### Rheology and texture analysis

The rheogram of the ME hydrogel composite was constructed using a rotational rheometer (Make-Anton Par, Model-RheolabQC) installed at CIF, Punjab University Chandigarh. Approximately 5 g of hydrogel composite was placed in a sample holder with a diameter of 9.995 mm and a length of 14.987 mm. The shear stress value was observed. The correlation of shear stress (τ) and shear rate (γ) was determined by a power law and Herschel–Bulkley model (Eqs. (1) and (2)).

1 $$\tau=\tau_o+{\mathrm{k}}{\gamma}^{\mathrm n}$$

2 $$\tau = {\mathrm{k}}\; \gamma^{\mathrm{n}}$$

where *k* is the consistency index (Pa sec^n^), *τ*_o_ is yield stress (Pa), and *n* is the power-law exponent.

The texture properties of ME hydrogel were determined by Texture Analyser™ (TA.XT plus Texture Analyser, Stable Microsystem, Surrey, UK). Twenty grams of sample was placed into the lower cone of the instrument. During the analysis, the upper cone probe penetrated the sample up to 2 mm above the sample holder surfaces. The test speed was maintained at 3.0 mm/s [30].

### In vitro antibacterial activity

The antibacterial activity of AzA, TTO, AzA with TTO combination (AzA-TTO), MET-6, and ME hydrogel composite formulations were studied against three bacterial strains, namely *S. aureus* (ATCC 29,213), *S. epidermidis* (MTCC 3382), and *P. acnes* (MTCC1951). Bacterial strains *S. aureus* and *S. epidermidis* were cultured in nutrient broth (NB) medium and incubated at 37 °C for 24 h. *P. acnes* were grown in brain heart infusion (BHI) medium under anaerobic conditions at 37 °C for 72 h [31].

#### Antibacterial agar well diffusion assay

Antibacterial activity was determined by using the agar well diffusion method. About 25 mL of media was solidified in a 100 mm sterile Petri dish. The bacterial culture of 0.2 McFarland Standard 600 nm was spread uniformLy on the prepared media plates using sterile cotton. The wells were punched with the cork borer (6 mm) in the agar, and different formulations were added to the separate wells. AzA, TTO, and AzA-TTO combination were diluted in 99.9% ethanol to have test concentration as 50 mg/mL for AzA, 50 mg/mL for TTO, and 50 mg/mL each for AzA-TTO combination. ME containing AzA 50 mg/mL and TTO 50 mg/mL loaded directly into the wells, while ME hydrogel composite was first diluted in the DMSO to have the test concentration as 50 mg/mL each for AzA and TTO. After the incubation of 24 h at 37 °C for *S aureus* and *S. epidermidis*, and 72 h at 37 °C for *P. acnes*, the zone of inhibitions was measured using HiAntibiotic Zone scale-C. Erythromycin was used as a positive control for *S. aureus* and *S. epidermidis* at a concentration of 1 mg/mL and *P. acnes* at 15 μg/mL [32].

#### Determination of MIC

The broth microdilution assay was carried out to determine the minimum inhibitory concentration (MIC) of AzA, TTO, AzA-TTO, MET-6, and ME hydrogel composite formulations for the different bacterial strains. The minimum concentration required to produce no visible growth of tested organisms was considered as MIC. To determine the MIC, 100 μL of each test formulation was taken in a flat-bottom 96-well microtiter plate containing 100 μL of NB for *S. aureus* and *S. epidermidis* BHI for *P. acnes*. Subsequently, each well pertaining to every test formulation was diluted in a ratio of 1:1 serially up to the 12th well. Ten microliters of bacterial culture suspension (1 × 10^8^ cells /mL) was added to each well. Controls viz. medium control and test control (AzA, TTO, AzA-TTO, ME, and ME hydrogel composite alone) wells were also reserved without any bacterial inoculums. Control for inoculums viability was also reserved, wherein no test formulation was added. The incubation was done for 24 h at 37 °C for *S aureus* and *S. epidermidis*, and 72 h for *P. acnes* at 37 °C, followed by resazurin dye (10 μL) color change was monitored visually. The growth was identified by the changes in color from purple to pink or colorless. The MIC is denoted by the minimum concentration of formulation at which the color changes from purple to pink. All samples were analyzed in triplicate [32].

### Ex vivo studies

#### Skin permeation studies

To conduct ex vivo permeation studies, a 2 cm^2^ Wistar rat skin was used as a diffusion membrane and placed on a diffusion cell. Wistar rats are among the most widely used strains in animal experimentations for gauging acne potential. The Wistar rats were sacrificed by spinal dislocation, and the hair was removed from the dorsal surface with the help of electric clippers. All the adhering adipose tissues were cleaned by employing sterile scissors from the skin before harvesting. The removed skin was then dipped into 0.9% normal saline for one hour to achieve equilibration before mounting the skin between the donor and receptor compartment of the diffusion assembly. The receptor compartment contained15 mL of diffusion medium, i.e., ethanol:phosphate buffer pH 6 (30:70). The receptor compartment was under continuous stirring with the help of a magnetic stirrer, and the temperature of the assembly was maintained at 32 °C by water-jacketed thermoregulators. 0.5 g of each formulation of AzA, i.e., MET-6, ME Hydrogel composite, and Aziderm (marketed formulation), were separately loaded into the donor compartment. 1 mL aliquot was collected from the receptor compartment and was replaced by 1 mL of fresh diffusion media to maintain sink condition. The sampling was done at 0.5, 1.5, 4, 6, 9, 12, and 24 h. The concentration of AzA in the donor compartment was analyzed by the validated RP-HPLC method, and the amount of cumulative AzA permeated in each time point was calculated using Eq. 3. The graph between cumulative amount permeated (μg/cm^2^) vs. time was also plotted [33].

3 $${Q_n} = {C_n}\, \times \,{V_0} + \sum\limits_{i = 1}^{n - 1} {C_i} \, \times \,{V_i}$$

*C*_*n*_ represents the amount of drug present in the receptor compartment at each time point of sampling, *C*_*i*_ denotes drug concentration of the *i*th sample, and *V0* and *Vi*, respectively, represent volumes of the solution in the receptor compartment and volume of the sample.

#### Skin retention studies

Skin mounted on the diffusion cell for permeation study was carefully removed upon completion of the permeation study, followed by cautiously removing adhered formulation. The drug content in the removed formulation was analyzed. The collected skin tissue was cleaned by washing it with ultrapure water thrice and dried on the lint-free cotton swab. Further, the skin was macerated in methanol using a tissue homogenizer followed by centrifugation at 7000 rpm to collect the supernatant. The drug was then extracted from the supernatant and analyzed by using RP-HPLC [34].

### In vivo studies

#### Skin compliance study

The skin compliance study was done in Wistar rats. The rats were grouped into two experimental and one control animals group containing three animals per group. All the animals were kept separately in individual cages and water, and food was supplied by ad libitum. The hairs on the skin surface of the dorsal side of the rats were removed by using a 0.1 mm animal hair clipper. The skin was then cleaned three to four times with a water-soaked cotton swab. Group 1 was maintained as control (without treatment), while groups 2 and 3 received ME hydrogel composite and marketed formulation (MKT gel), respectively. After receiving the treatment once a day for two weeks to the experimental and the control, the remaining formulation was removed from the skin by a dry cotton swab followed by a wet cotton swab. After 1 h of cleaning, a photograph of the treated skin areas was taken with the help of a digital camera. Subsequently, the animals were sacrificed, and the animals’ skin was processed with hematoxylin and eosin stain to examine the histopathological changes microscopically. [35].

#### Anti-acne activity: testosterone-induced acne model

Male Swiss albino mice having bodyweight of 20–30 g were separated into four groups. Each group contains three mice. In groups 2–4, 2% ethanolic solution of testosterone (TS) was applied to induce acne in the skin of the dorsal side of the animal, while group 1 was served as the control group. Acne started appearing after 2 weeks, and subsequently, its treatments were started using different azelaic acid formulations (0.1 g per 4 cm^2^) once a day for the next two consecutive weeks. The control group (group 1) received normal saline as a placebo, whereas group 2 were maintained without treatment as disease control, and groups 3 and 4 were treated with ME hydrogel composite and MKT gel, respectively. Visual observation was done in regular intervals to monitor the presence of papules or other visible changes. The anti-acne potential was calculated as a function of reduction in the papule density/4 cm^2^ area after treatment. Upon completion of 2-week treatment, the animals were sacrificed, and the skin was harvested. Each control and treated skin sample was fixed in 10% formalin, embedded in paraffin, and sectioned transversely. The skin sections were processed and stained with hematoxylin and eosin, followed by microscopic study. The changes in sebaceous gland number and size were noted [35].

## Results and discussion

### Preparation of ME

#### Screening of oils, surfactants, and co-surfactants

All the excipients selected for ME preparation were certified under the generally regarded as safe (GRAS). Determination of the solubility profile of the drug in the various excipients is a critical aspect. The selection of safe, non-irritant, or non-toxic surfactant, higher CMC of nonionic surfactant than the ionic counterparts, choice of surfactants with hydrophilic-lipophilic balance (HLB) values that leads to the spontaneous formation of a clear and transparent microemulsion was also considered. AzA was best soluble in IPM, and it was utterly miscible with TTO also. All the surfactants studied AzA dissolved maximally in nonionic surfactants, i.e., Labrasol and Tween 80. Mixtures of PL-90G: ethanol (1:10) and Transcutol P were selected as cosurfactants and reported excellent permeation-enhancing properties. The phospholipidic nature of PL-90G enhances the skin permeability of the drug as phospholipids get readily absorbed in all biological membranes [20].

#### Construction of pseudo-ternary phase diagram

**Fig. 1:**
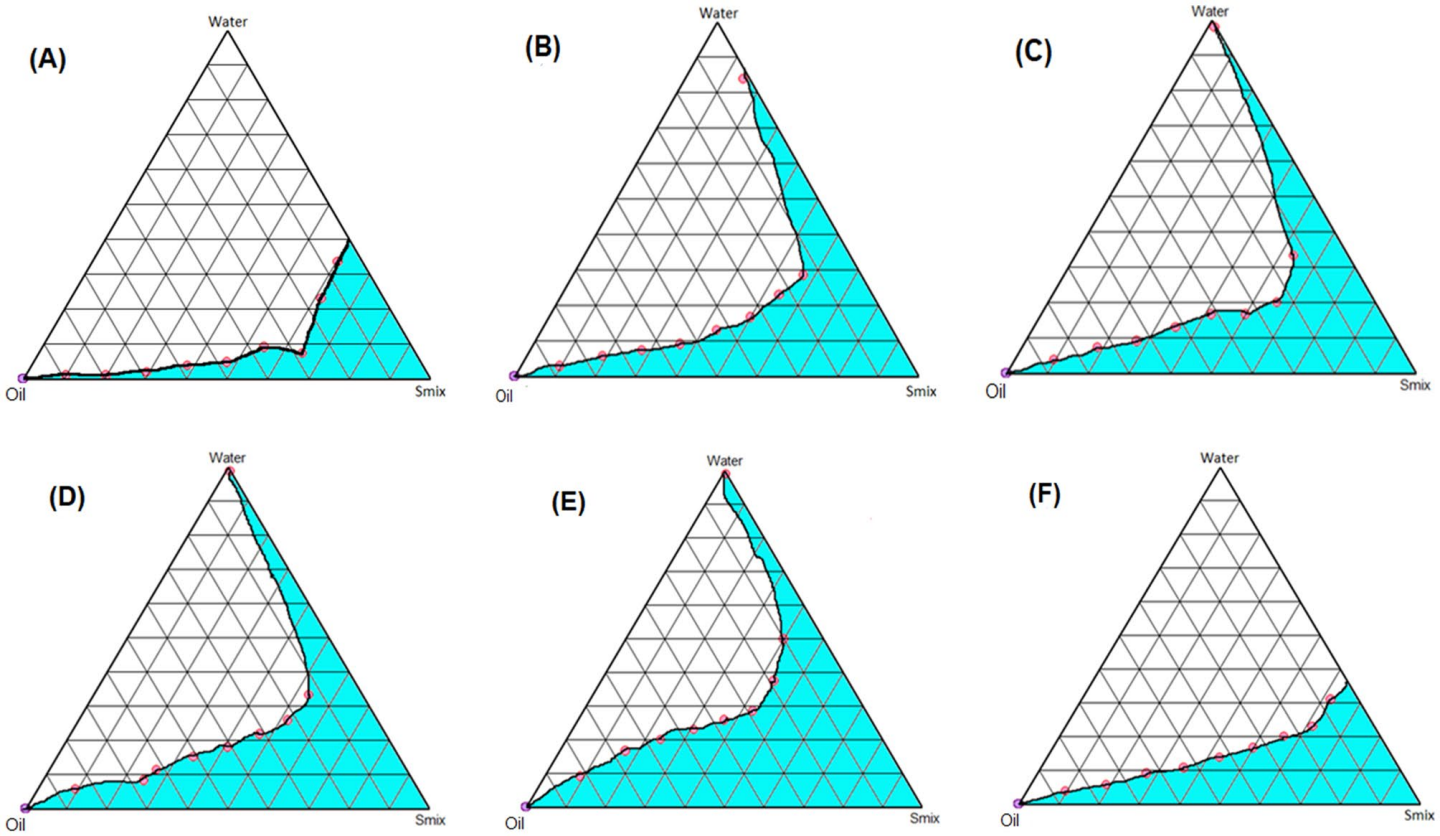
Pseudo-ternary phase diagrams of MEs composed of oil (IPM), Smix (Labrasol: CS (Transcutol P: (PL-90G: Ethanol (1:10))and water at various Smix ratios: **A** 1:1, **B** 2:1, **C** 3:1, **D** 4:1, **E** Smix (Labrasol), **F** Smix (Labrasol:Transcutol (1:1)

**Fig. 2:**
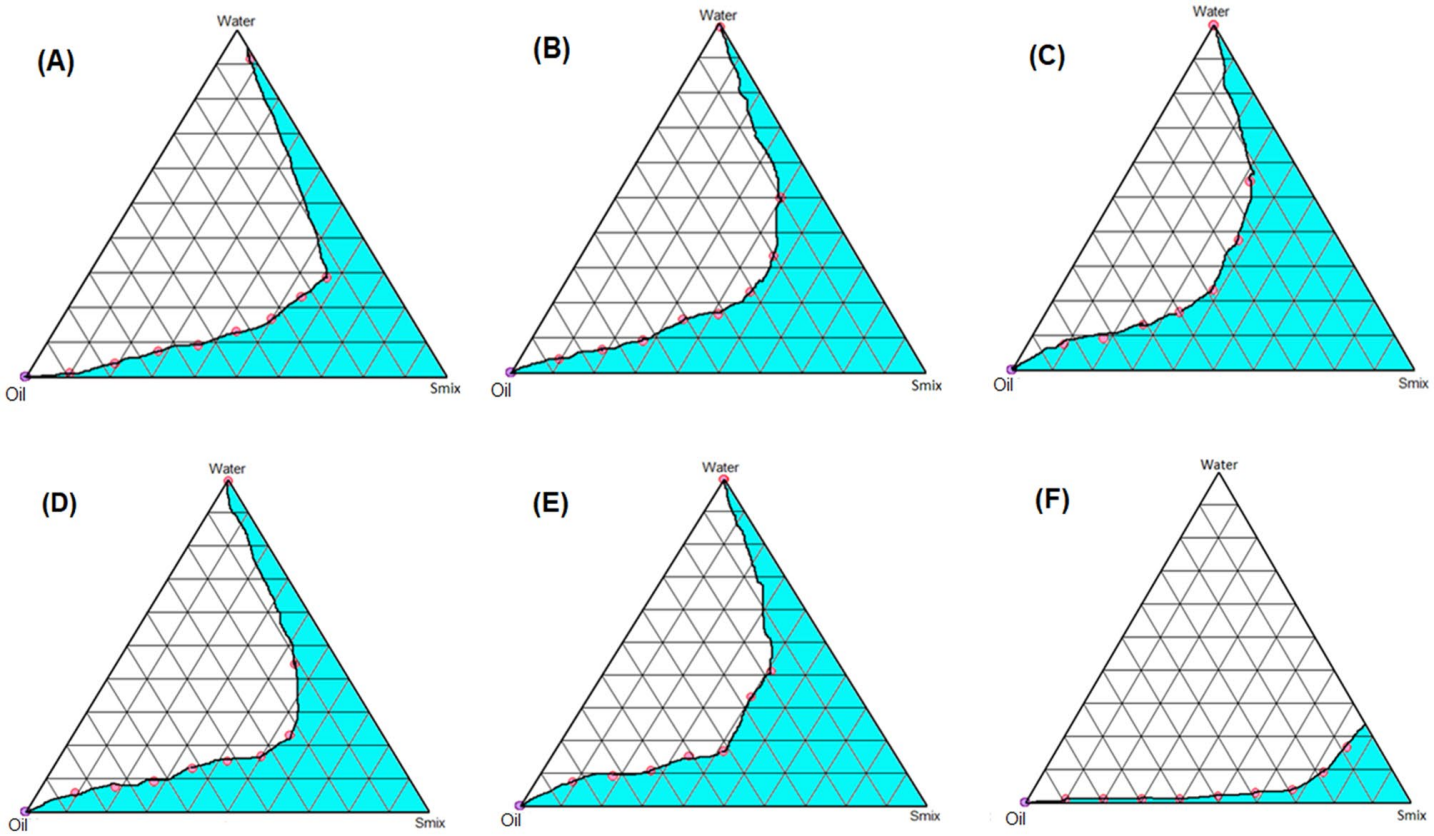
Pseudo-ternary phase diagrams of MEs composed of oil (IPM), Smix (Tween 80: CS (Transcutol P: (PL-90G: Ethanol (1:10)) and water at various Smix ratios: **A** 1:1, **B** 2:1, **C** Smix (Tween 80:PL-90G:ethanol) (1:10), **D** Smix (Tween 80:Transcutol P) (1:1), **E** Smix (Tween 80:Transcutol P) (2:1), **F** Smix (Transcutol:PL-90G) (1:10)

In order to achieve the optimum composition of ME, the PTPDs were constructed for various Smix ratios (Table 1). In general, out of the two different surfactants studied, Tween 80 yielded a better ME area than Labrasol (Figs. 1 and 2). On increasing the Labrasol concentration in the Smix ratio from 1:1 to 2:1 (Fig. 1A and B), ME area considerably increased due to the better emulsifying ability of Labrasol. However, there was not much difference in the ME areas of Smix 2:1, 3:1, and 4:1 (Fig. 1B, C, D). In PTPD, constructed by taking Labrasol as Smix without any CS yielded bigger ME area (Fig. 1E) [26]. Whereas ME area PTPD constructed employing Labrasol and Transcutol P as Smix in 1:1 ratio, was smallest. Overall, the presence of CS in the Smix with Labrasol did not contribute to enhancing the emulsifying ability of Labrasol. In the PTPDs constructed employing Tween 80, the best ME area was obtained when Tween 80 in a combination of PL-90G and ethanol was taken as Smix (Fig. 2C). While adding Transcutol P in CS resulted in a decrease in ME area (Fig. 2A, B). PTPD constructed employing Transcutol P and PL-90G:ethanol as CS with Tween 80 (surfactant) as Smix ratio 1:1 yielded relatively smaller ME area vis-a-vis 2:1 Smix ratio. This could be attributed to the better emulsifying ability of Tween 80 in composition to Transcutol P. The smallest area was obtained when no Tween 80 was employed (Fig. 2F).

#### Selection of formulations based on the obtained phase diagram

The main criteria for solubility of AzA and TTO were selecting various ME compositions from the ME area (Table 2). The ME compositions having AzA concentration of more than 50 (mg/mL) were considered. However, out of two compositions, in each PTPD, the ME composition having higher drug solubility was taken for further characterization study (Table 3).

**Table 3:** Droplet size and PDI of formulation

**S. no**	**Sample ID**	**S**_**mix**_**(%)**		**Oil (%)**	**Water (%)**	**Droplet size (nm)**	**PDI**
		**Ratio (S:CoS)**	**Qty**				
1	MET-1	Lab:CoS (2:1)	55	40	5	950.1	0.79
2	MET-2	Lab:CoS (3:1)	55	40	5	610.1	0.29
3	MET-3	T-80:CoS (1:1)	60	35	5	664.8	0.01
4	MET-4	T-80:CoS (2:1)	50	45	5	424.0	0.56
5	MET-5	T-80:Trans (1:1)	60	35	5	560	0.39
*6*	*MET-6*	*Lab:CoS (4:1)*	*60*	*35*	*5*	*357.4*	*0.64*

*CoS* Transcutol P:( PL-90G:ethanol (1:10)), *Trans* Transcutol P, *Lab* Labrasol

### Characterization of AzA-TTO-loaded MEs (MET1-6)

**Fig. 3:**
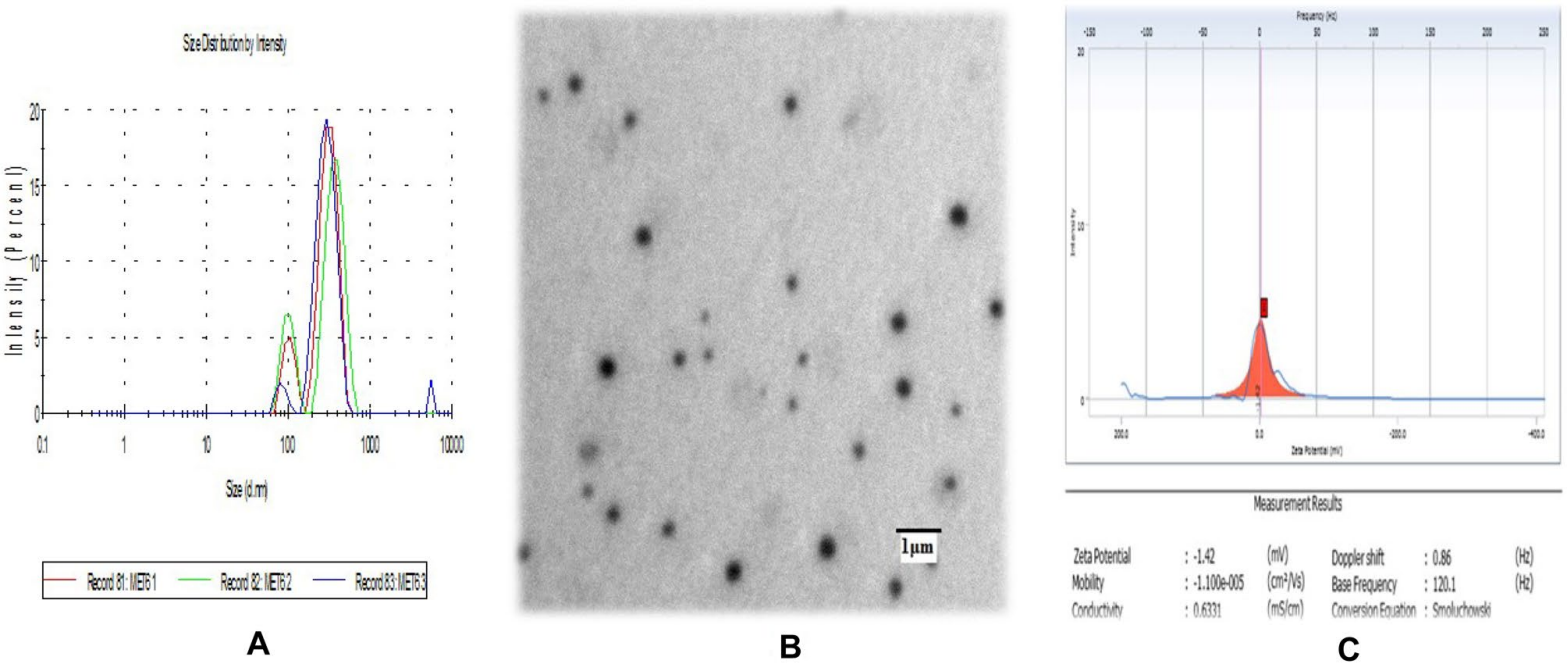
**A** Particle size distribution of AzA-loaded ME. **B** TEM image of optimized ME formulation (magnification 200,000 ×). **C** Zeta potential of optimized AzA-ME dispersion

The droplet size with all the selected ME formulations ranged between 357 to 950 nm, as depicted in Table 3. A consistent decrease in the particle size of the MEs can be correlated with the increase in Smix concentration. This might be due to the decrease in surface tension due to high surfactant concentration, which possibly stabilizes the developed surfaces of the smaller particle size. The PDI refers to the homogeneity of the colloidal dispersion. The particles size ranged between 357 to 950 nm, while all PDI values were less than 1, indicating the homogeneity of the colloidal dispersions. Among the different ME formulations, MET-6 was selected based on its small particle size (357.4 nm ± 5%). The PDI graph of MET-6 is also depicted in Fig. 3A.

A high value of transmittance (> 90%) was observed for MET-6, confirming the optical clarity/transparency of the formulation, which is a prerequisite for MEs. The high transmittance value was due to the highest surfactant concentration in the MET-6 formulation. The MET-6 was also examined by microscopically TEM (Fig. 3B) (magnification 200,000 ×), revealing the spherical topography with no aggregation. The zeta potential of MET-6 was found −1.42 mV (Fig. 3C), which was close to zero. It implied that stability was achieved possibly due to the steric repulsion of the nonionic surfactant molecules in this system and not the electrostatic repulsion of the particles [36]. However, it has been reported that the stability of nonionic surfactants containing emulsions and ME is independent of their zeta potential values [37].

### Incorporation of ME in secondary topical vehicle

#### Organoleptic properties

**Fig. 4:**
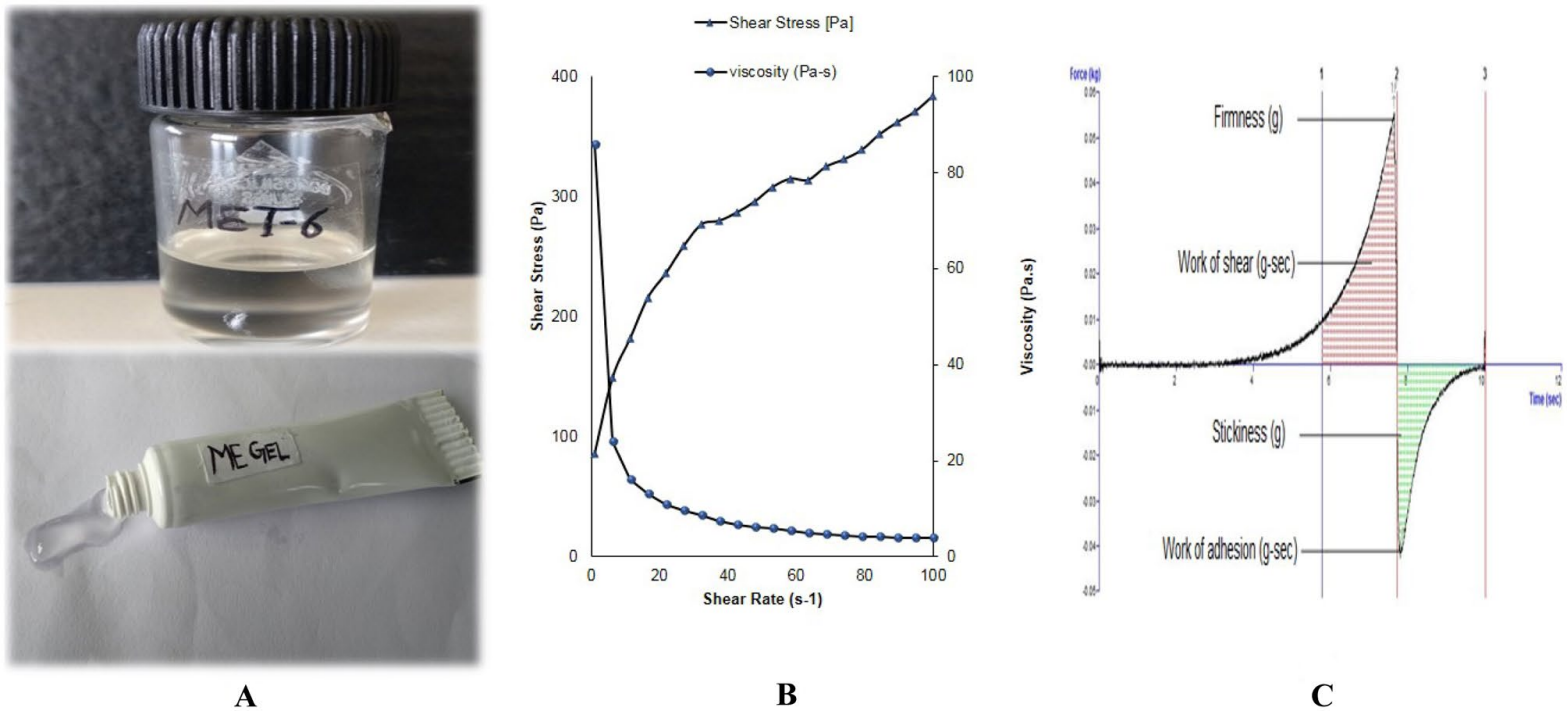
**A** Photomicrograph showing the images of ME formulation and ME hydrogel composite formulation. **B** Plots between shear rate vs. viscosity and shear rate vs. shear stress for optimized ME hydrogel composite. **C** Textural analysis of AzA-ME gel

All AzA-loaded MEs were found to be transparent and colorless (Fig. 4A). Phase separation or creaming was absent in the prepared MEs. Both the optimized ME and ME hydrogel composite formulation exhibited a smooth feel without any grittiness or tackiness.

#### Rheology and texture analysis

Rheological and texture characteristics of optimized ME hydrogel composite are shown in Table 4. In a rheological study of the ME hydrogel composite, the value of *n* was less than 1, which suggests the system follows pseudo-plastic behavior that indicates shear thinning behavior (Fig. 4B). The viscous hydrogel is composed of long-chain molecules hooked and entwined at a static or low flow rate. The scattered chains are rolled and contracted into a group on increasing shear rate, thereby reducing mutual hooking and the apparent viscosity, which clues to the shear-thinning phenomenon [38].

**Table 4:** Rheological and texture characteristics of AzA and TTO-loaded ME hydrogel composite

Formulation parameter	ME hydrogel composite
*n*	0.435
*k* (Pa)	46.43
yield value (Pa)	86.54
Viscosity (Pa-s)	10.73
Firmness (g)	55.832
Work of shear (g-s)	52.725
Stickiness (g)	−42.709
Work of adhesion(g-s)	−26.800

*n* power-law exponent, *k* consistency index

From the force–time curve plotted, textural parameters of cohesiveness [i.e., cycle 2 (area of work during the second compression) divided by cycle 1 (area of work during the first compression)], adhesiveness (i.e., a total negative area in cycle one denoting the work required to pull the compressing probe away from the sample), spreadability (work of shear), forward extrusion (firmness), stickiness (area of the negative force curve), were acquired.

The yield value obtained for the gel was high, which depicts the rigidity of the gel system. The yield value obtained for the gel was 86.54, firmness (55.832 g), work of shear (52.725 g^−s^), work of adhesion (−26.800 g^−s^), and stickiness (−42.709 g) (Table 4). The prepared ME hydrogel system showed adequate cohesiveness, essential for the formulation to retain at the application site (Fig. 4C). These parameters further predict the appreciable mechanical strength, easy applicability, ease of extrusion from the tube of the formulation, which are prerequisites for an ideal topical hydrogel preparation.

### In vitro antibacterial activity

#### Analysis of the antibacterial activity of AzA, TTO, AzA and TTO, ME and ME hydrogel composite formulations

**Fig. 5:**
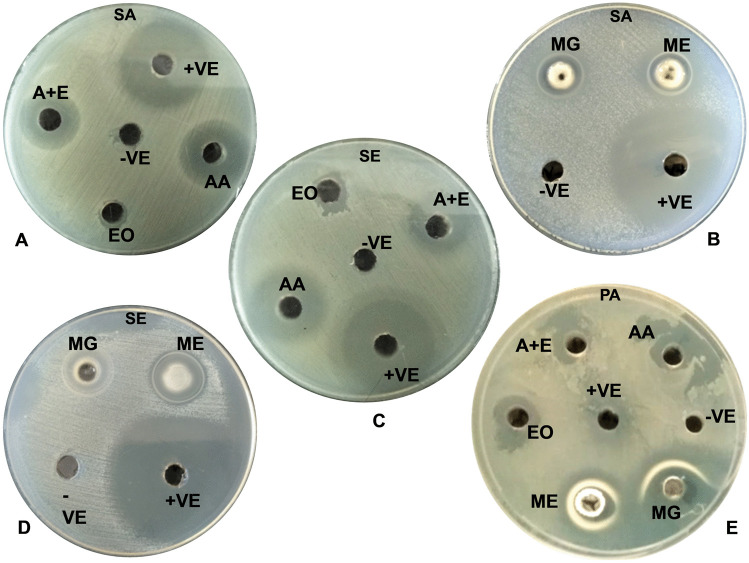
Antimicrobial activity of AzA and EO of plant *Melaleuca alternifolia* (TTO), ME, and ME hydrogel composite formulations against *S. aureus*, *S. epidermidis*, and *P. acnes*. **A** *S. aureus* (SA) (AA)–azelaic acid 50 mg/ml in ethanol, (EO)—TTO 50 mg/ml in ethanol, (A + E)–azelaic acid + TTO 50 mg/ml + 50 mg/ml, (−ve)–ethanol and (+ ve)–erythromycin (1 mg/ml). **B** *S. aureus* (SA) (ME)–microemulsion 50 mg/ml, (MG)–ME hydrogel composite 50 mg/ml in DMSO, (−ve)–DMSO and (+ ve)–erythromycin (1 mg/ml). **C** *S. epidermidis* **(**SE) (AA)–azelaic acid 50 mg/ml in ethanol, (EO)–essential oil 50 mg/ml in ethanol, (A + E)–azelaic acid + essential oil 50 mg/ml + 50 mg/ml in ethanol, (−ve)–ethanol and (+ ve)–erythromycin (1 mg/ml). **D** *S. epidermidis* (SE) (ME)–microemulsion 50 mg/ml, (MG)—ME hydrogel composite 50 mg/ml in DMSO, (−ve)–DMSO and (+ ve)–erythromycin (1 mg/ml). **E** *P. acnes* (PA), (AA)–azelaic acid 50 mg/ml in ethanol, (EO)–TTO 50 mg/ml in ethanol, (A + E)–azelaic acid + TTO 50 mg/ml + 50 mg/ml, (ME)–microemulsion 50 mg/ml, (MG)–ME hydrogel composite 50 mg/ml in DMSO, (−ve)–DMSO and (+ ve)–erythromycin (15 μg/ml)

The diameters of zones of bacterial inhibition against various test formulations are depicted in Table 5 and Fig. 5. Ethanol and DMSO, taken as a negative control, did not show any activity, whereas erythromycin employed as + ve control demonstrated excellent antimicrobial activity against the tested microorganism. AzA also showed strong antibacterial activity against *P. acnes*, *S. aureus*, and *S*. *epidermidis*. Surprisingly, TTO alone did not exhibit any activity against all the test organisms; this could be because TTO is ineffective at low concentrations (50 μg/mL). However, in combination with AzA revealed appreciable activity, it may be due to the synergistic bactericidal effect of TTO and AzA on the cells, owing to the keratolytic effect of AzA. While the activity of AzA and TTO loaded ME was comparable to AzA against *S. aureus*, *S. epidermidis*, however, AzA-TTO combination was significantly better in the case of *P. acnes* compared to AzA alone. Further AzA and TTO loaded ME demonstrated better zone of inhibition vis-a-vis all the other formulations against all the tested organisms (≈23 ± 1.0 mm) as they interacted with the bacterial cell wall, enhanced cellular permeability, and reduced cellular permeability hydrophobicity of bacterial cells, and decreased the viability of the bacterial cells.

**Table 5:** Diameters of zones of bacterial inhibition obtained using AzA, TTO, AzA, and TTO, ME and ME hydrogel composite formulations

Formulations	Concentration	Vol. used	Diameter obtained for the zone of inhibition, σ ± SD (mm) (amount of antibacterial tested (mg/mL))		
			ATCC 29,213 (*S. aureus*)	MTCC 3382 (*S. epidermidis*)	MTCC1951 (*P. acnes*)
AzA	50 mg/mL	80 μL	21.3 ± 0.5	20.6 ± 1.1	11.3 ± 0.5
EO	50 mg/mL	80 μL	ND	ND	12.3 ± 0.5
AzA with EO	50 + 50 mg/mL	80 μL	15.3 ± 1.5	16 ± 1.0	ND
ME	50 mg/mL	80 μL	23 ± 1.0	23.6 ± 1.5	22.3 ± 0.5
Hydrogel Composite	50 mg/mL	80 μL	19 ± 1.7	19.3 ± 0.5	18.3 ± 0.5
Erythromycin (+ ve)	1 mg/mL	10 μL	23 ± 1.0	29 ± 1.0	12 ± 0
Ethanol (−ve)	-	80 μL	ND	ND	ND
DMSO (−ve)	-	80 μL	ND	ND	ND

*ND* no zone of inhibition detected; the zone of inhibition values are an average from two independent experiments, *EO* tea tree oil, *AzA* azelaic acid

#### Minimum inhibitory concentration (MIC) of AzA, TTO, AzA and TTO combination, ME and ME Hydrogel composite formulations against bacterial strains

The order of MIC values (Table 6) against *S. aureus* was observed to be as follows*:* TTO (3.12 mg/mL) > AzA ≈ AzA with TTO ≈ ME hydrogel composite (0.78 mg/mL) > ME (0.19 mg/mL) > erythromycin (0.02 mg/mL). For *S. epidermidis* MIC values were found to be as follows: TTO (6.25 mg/mL) > ME hydrogel composite (3.12 mg/mL) > AzA≈AzA with TTO *(*0.78 mg/mL) > ME (0.39 mg/mL) > erythromycin (0.02 mg/mL). And for *P. acnes* MIC value were found to be EO (6.25 mg/mL) > AzA ≈ ME (3.12 mg/mL) > AzA with TTO (1.56 mg/mL) > ME hydrogel composite (0.39 mg/mL). Although the antibacterial effect of TTO is nonsignificant, AzA-loaded TTO ME showed significantly lower MIC values compared with TTO alone in this present investigation. Further, ME hydrogel composite containing AzA and TTO revealed the lowest MIC value (0.39 mg/mL) against *P. acnes* compared to AzA and TTO. The superior efficacy of ME hydrogel could be ascribed to the synergistic activity of the AzA and TTO in the form of ME hydrogel, better interaction with the bacterial cell wall, followed by increased skin contact time, and sustained drug release.

**Table 6:** MIC values for azelaic acid, EO (TTO), AzA and EO combination, ME and ME hydrogel composite formulation against different test organisms

**Formulations**	**MIC (mg/mL)**		
	**ATCC 29,213 (*S. aureus*)**	**MTCC 3382 (*S. epidermidis*)**	**MTCC1951 (*P. acnes*)**
AzA	0.78	0.78	3.12
EO	3.12	6.25	6.25
AzA with EO	0.78	0.78	1.56
ME	0.19	0.39	3.12
ME hydrogel composite	0.78	3.12	0.39
Erythromycin (+ ve)	0.02	0.02	-

*EO* tea tree oil, *AzA* azelaic acid, *MIC* minimum inhibitory concentration

### Ex vivo studies

#### Skin permeation studies

**Fig. 6:**
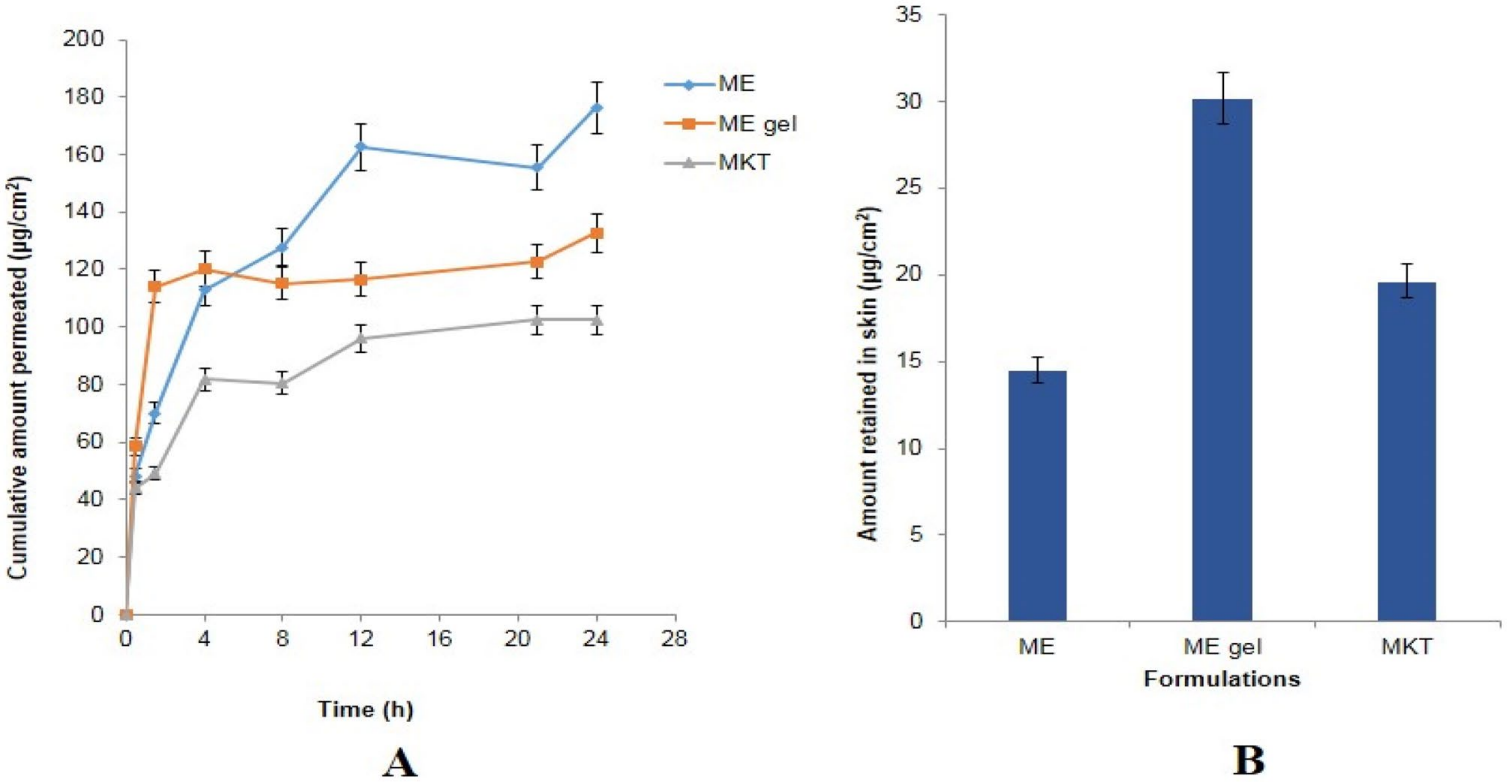
**A** Ex vivo skin permeation profiles of AzA from the ME (microemulsion), ME hydrogel composite (microemulsion gel), and the MKT (marketed formulation). **B** Plot showing the skin retention values of ME (microemulsion), ME hydrogel composite (microemulsion gel), and MKT (marketed formulation)

The ex vivo skin permeation and penetration study conducted with the various formulation is shown in Fig. 6A. Cumulative amount permeated (± SD) after 24 h of marketed formulation 10%w/w Aziderm® gel (102.49 μg/cm^2^) < ME hydrogel composite (132.72 μg/cm^2^) < optimized ME (176.30 μg/cm^2^). Higher permeation values could be ascribed to the use of phospholipid and Transcutol P, for the formulation, which lowers the surface tension between the ME and stratum corneum (SC) and allows better contact and penetration into SC by surfactants and the associated drug. As the globule size of ME formulation is smaller, it provides a larger surface area followed by better percutaneous absorption. Percutaneous absorption is also facilitated due to permeation enhancers such as phospholipon 90G and Labrasol in the MEs, as reflected in the results of the skin permeation study.

#### Skin retention studies

The amount AzA retained in the skin from the different formulations is shown in Fig. 6B. The skin retention observed the following order: ME hydrogel composite (30.16 μg/cm^2^) > Aziderm® (19.64 μg/cm^2^) > AzA-ME (14.52 μg/cm^2^). Higher retention vis-à-vis marketed formulation could be attributed to the ability of ME hydrogel composite to form the reservoir in the layers more effectively also the formation of more close contact of the drug with skin lipids and thereby drug retention in the skin layer [30].

### In vivo studies

#### Skin compliance study

**Fig. 7:**
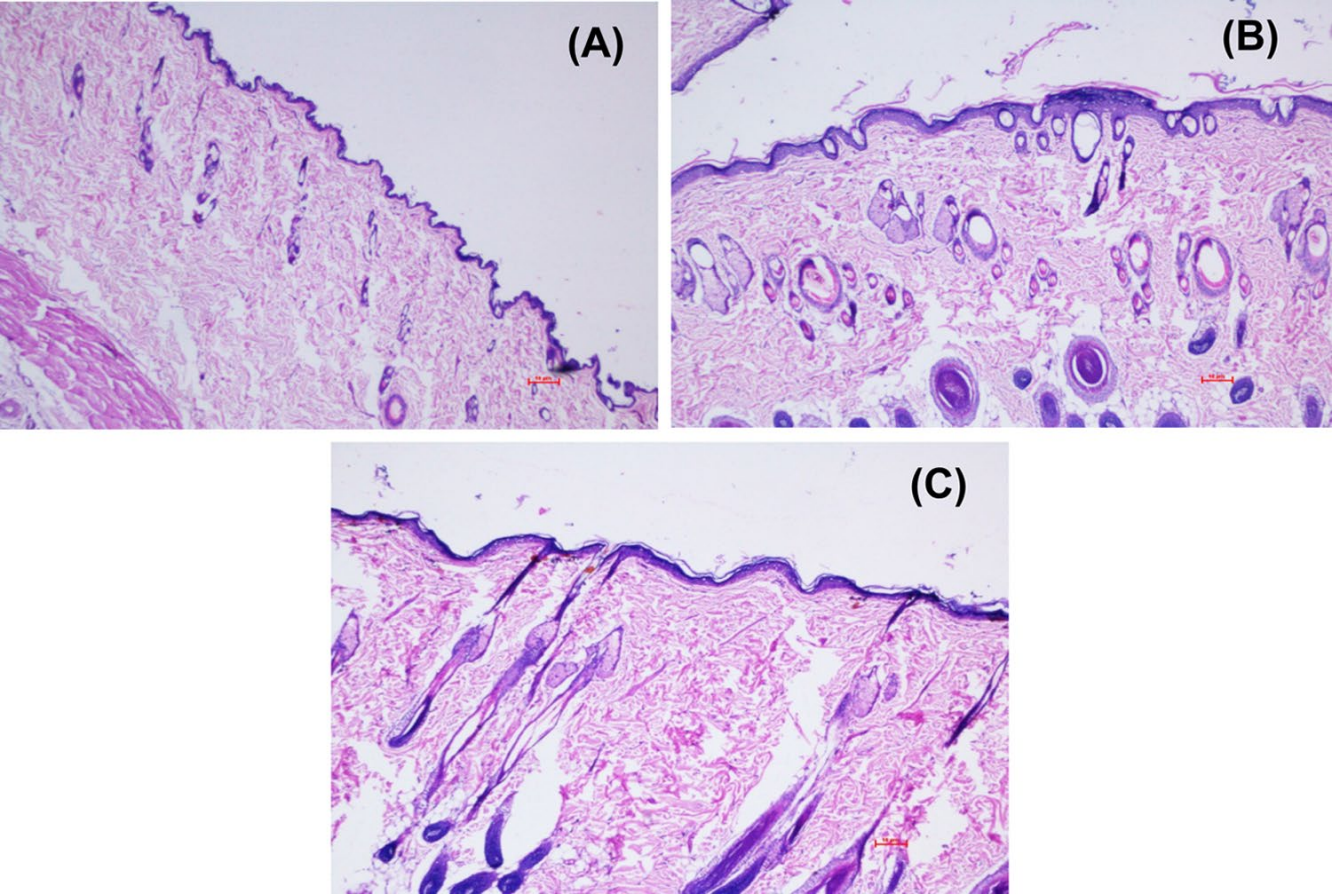
Photomicrographs of skin histology sections treated with **A** control, **B** ME treated, and **C** MKT formulation

Figure 7 shows the photomicrographic representation of rat skin surface after treating it with normal saline (control), ME hydrogel composite, and MKT gel. The histology of the control-treated group revealed a normal intact skin with a multilayer of epidermis, dermis, and the subcutaneous fatty layer was recorded. Slight changes were observed in the ME-treated skin without any change in the dermal layer. The MKT formulations treated skin showed no significant changes in the normal histology, confirming the biocompatibility of the formulations. The ME-treated rat skin SC layer became thicker without any apparent change in epidermis and dermis (Fig. 7C), whereas in gel application, all the SC, epidermis, and dermis layers remained intact. The addition of Carbopol® 934 increased the viscosity of the prepared ME gel by generating a three-dimensional network structure. This network structure of the prepared gel further reduced the chances of direct contact of AzA with the skin layers and probably reduced the chances of any such adverse effects. In addition, the skin treated with ME hydrogel composite (Fig. 7B) and MKT gel showed no sign of inflammation cells, confirming better tolerability of the ME-based formulation. This may be due to the drug encasement within the biocompatible components of ME, viz., phospholipid and surfactant.

#### Anti-acne activity: testosterone induced acne model

**Fig. 8:**
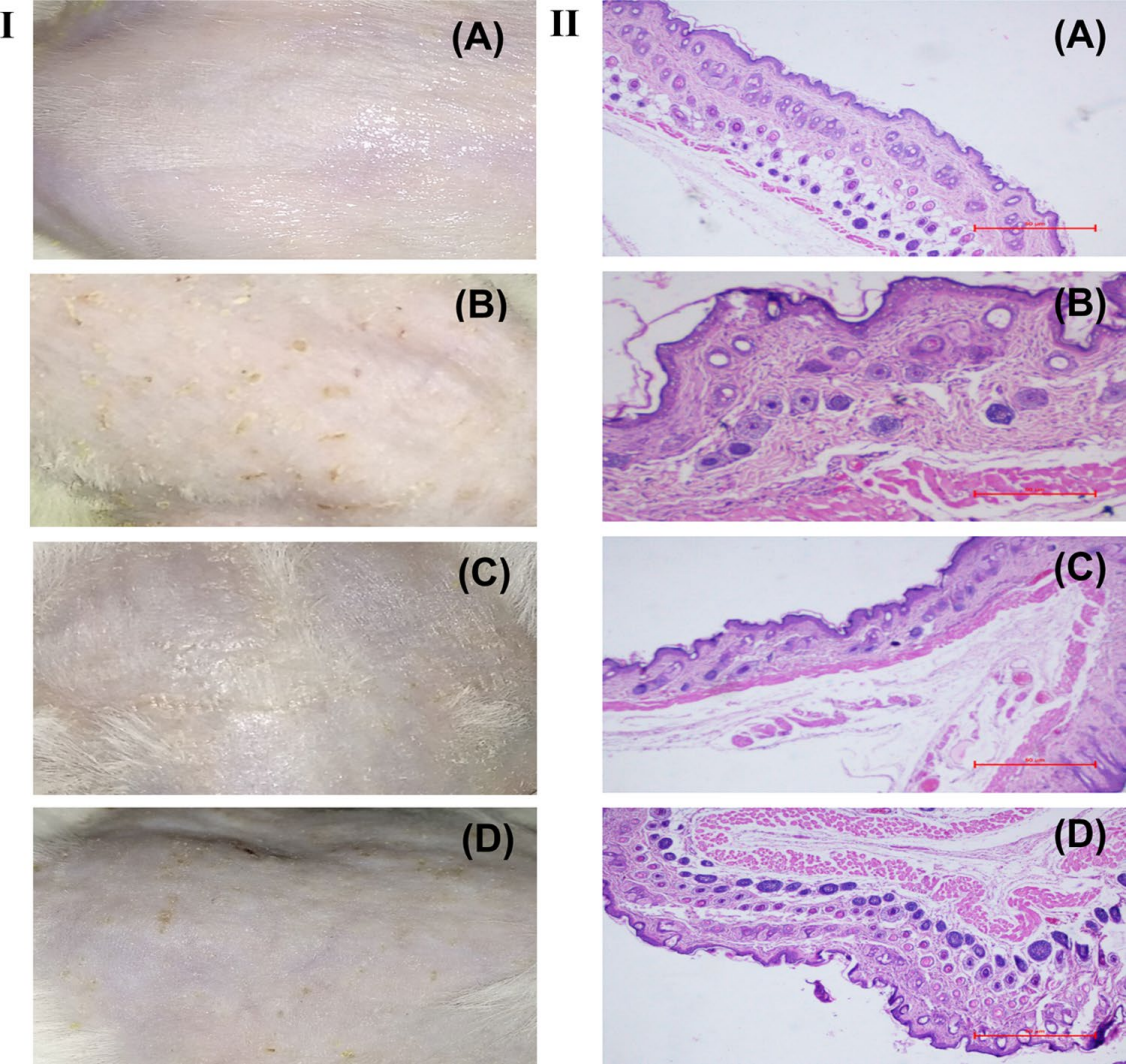
**A**, **B** Evaluation of mice skin after 4th week of once a day application of control (normal saline). **A** Macroscopic image. **B** Microscopic image. **C**, **D** Evaluation of mice skin after 4th week of once a day application of testosterone (for the first 2 weeks) and then no treatment (disease control). **C** Macroscopic image. **D** Microscopic image. **E**, **F** Evaluation of mice skin after 4th week of once a day application of testosterone (for the first 2 weeks) and ME Hydrogel composite (for subsequent weeks). **E** Macroscopic image. **F** Microscopic image. **G**, **H** Evaluation of mice skin after 4th week of once a day application of testosterone (for the first 2 weeks) and MKT gel (for subsequent weeks). **G** Macroscopic image. **H** Microscopic image

Figure 8 shows considerable changes in the skin after treating it with AzA formulations. The changes visible in animal skin further depicts that application of TS developed severe acne in the animals after 2 weeks, which reduced considerably after the treatment with AzA formulations (Fig. 8). Figure 8 gives evidence of a significant reduction in papule density in the animals treated with AzA in comparison to the untreated group as observed visually. The mean percentage reductions in papule density were in order of MKT gel (72.69 ± 4.67%) < ME hydrogel composite formulation (93.75 ± 1.64%). The histological evaluation gave pieces of evidence of sebaceous hyperplasia (i.e., increase in size and number of sebaceous glands) and follicular hyperkeratosis in pilosebaceous units, which indicates the development of acne (Fig. 8B). On the other side, treatment with AzA showed a significant reduction in the lesion, sebaceous gland hyperplasia, and seborrhea (Fig. 8C), whereas MKT gel treated skin shows mild sub-epidermal inflammation. Surprisingly, the ME hydrogel composite skin histology was similar to the normal skin showing a significant reduction in papule density and acne lesions. In addition, a group that received ME hydrogel composite did not show any evidence of skin peeling and desquamation, thereby showing better skin tolerance and minimum toxicity concerning the MKT formulation. The release of AzA in a sustained manner with increased skin permeability and the synergistic activity of TTO could be the possible reasons for improved therapeutic efficacy and increased tolerability of ME Hydrogel composite vis-a-vis MKT gel.

## Conclusion

AzA and TTO loaded ME and ME hydrogel composite formulations were successfully developed and evaluated for in vitro, in vivo, and ex vivo efficacy and safety. Developed ME hydrogel composite formulations protected the direct exposure of the drug to the skin, thereby reducing the side effects and have the best skin permeation and retention characteristics vis-à-vis marketed formulation. In vitro antibacterial efficacy of the developed ME hydrogel composite systems revealed a better zone of inhibition and low MIC values against *S. aureus*, *S. epidermidis*, and *P. acne*. The findings are of considerable significance keeping in view the dose-dependent adverse effects of AzA. Stability studies for ME formulation and ME hydrogel composite will be conducted in future work. Additionally, clinical studies can be done to evaluate the potential of the combination of AzA and TTO in the form of ME hydrogel.

## Data Availability

All data can be made available upon request.

## Abbreviations

AzA: Azelaic acid
TTO: Tea tree oil
ME: Microemulsion
MIC: Minimum inhibitory concentration
PL-90G: Phospholipipon 90G
DMSO: Dimethyl sulfoxide
TEA: Triethanolamine
BHI: Brain heart infusion
CS: Co-surfactant
PTPD: Pseudo-ternary phase diagram
EO: Tea tree oil
HLB: Hydrophilic lipophilic balance
CAN: Acetonitrile
A: *P. acnes*
SA: *S. aureus*
SE: *S. epidermidis*
CPCSEA: Committee for the purpose of control and supervision of experiment on animals
SC: Subcutaneous
MKT: Marketed formulation
TS: Testosterone
